# Bibliometric Analysis on COVID-19: A Comparison of Research Between English and Chinese Studies

**DOI:** 10.3389/fpubh.2020.00477

**Published:** 2020-08-14

**Authors:** Jingchun Fan, Ya Gao, Na Zhao, Runjing Dai, Hailiang Zhang, Xiaoyan Feng, Guoxiu Shi, Jinhui Tian, Che Chen, Brett D. Hambly, Shisan Bao

**Affiliations:** ^1^School of Public Health, Gansu University of Chinese Medicine, Lanzhou, China; ^2^Center for Evidence-Based Medicine, Gansu University of Chinese Medicine, Lanzhou, China; ^3^Evidence-Based Medicine Center, School of Basic Medical Sciences, Lanzhou University, Lanzhou, China; ^4^Key Laboratory of Evidence-Based Medicine and Knowledge Translation of Gansu Province, Lanzhou, China; ^5^School of Public Health, Lanzhou University, Lanzhou, China; ^6^School of Clinical Medicine, Gansu University of Chinese Medicine, Lanzhou, China; ^7^Discipline of Pathology, Charles Perkins Centre, Faculty of Medicine and Sciences, School of Medical Sciences, The University of Sydney, Sydney, NSW, Australia

**Keywords:** bibliometric, COVID-19 outbreaks, SARS-CoV-2, English, Chinese

## Abstract

**Background:** As an emerging infectious disease, COVID-19 has garnered great research interest. We aimed to explore the differences between English language and Chinese language Medical/Scientific journals publications, particularly aiming to explore the efficacy/contents of the literature published in English and Chinese in relation to the outcomes of management and characterization of COVID-19 during the early stage of COVID-19 pandemic.

**Methods:** Publications on COVID-19 research were retrieved from both English and Chinese databases. Bibliometric analyses were performed using VOSviewer 1.6.14, and CiteSpace V software. Network maps were generated to evaluate the collaborations between different authors, countries/provinces, and institutions.

**Results:** A total of 143 English and 721 Chinese original research articles and reviews on COVID-19 were included in our study. Most of the authors and institutions of the papers were from China before March 1st, 2020, however, the distribution of authors and institutions were mainly in developed countries or more wealthy areas of China. The range of the keywords in English publications was more extensive than those in Chinese. Traditional Chinese Medicine was seen more frequently in Chinese papers than in English. Of the 143 articles published in English, 54 articles were published by Chinese authors only and 21 articles were published jointly by Chinese and other overseas authors.

**Conclusions:** The publications in English have enabled medical practitioners and scientists to share/exchange information, while on the other hand, the publications in the Chinese language have provided complementary educational approaches for the local medical practitioners to understand the essential and key information to manage COVID-19 in the relatively remote regions of China, for the general population with a general level of education.

## Introduction

The seriousness of the rapid spread of the SARS-CoV-2 virus has caused people to panic around the world since December 2019 (1). The tremendous danger of SARS-CoV-2, with a basic reproduction number (*R*_0_) ranging from 2.30 to 3.58 (2), resulting in a pandemic with the number of infections reaching 9,653,048 to date (3). Consequently, considerable attention has been focused on COVID-19 from medical practitioners/scientists around the world to inhibit/stop the continuous transmission of SARS-CoV-2 and to develop guidelines for the effective treatment of severe cases.

To fight SARS-CoV-2, authorities in many countries have enforced social isolation restrictions to control the epidemic of COVID-19 throughout their countries, strategies utilized in China include wearing of facemasks in public areas, and minimizing outdoor, particularly mandating no public and/or private social gatherings (4, 5). The internet classes has allowed schools to continue to educate without classroom (6). Consequently, newly identified local COVID-19 cases have been reduced to near 0 in all of the provinces in China (7), mainly due to the active approaches outlined above, and strict limits to interstate/international travel. However, outside China, many countries now face escalating epidemics and are feeling overwhelmed due to the highly contagious nature of COVID-19.

As an emerging infectious disease, COVID-19 has garnered great research interest. Medical practitioners/scientists are studying the disease from various scientific and clinical areas, including specialists in infectious diseases, virology, microbiology. Many uncertainties remain as to certain epidemiological, seroepidemiological, clinical and virological characteristics of the virus and associated clinical features. The key task is to explore how to enhance host defenses and/or destroy viral resistance (8). Many researchers have published their data within top international, peer-reviewed, highly reputable journals, including NEJM, Lancet, Nature and Science (9). There are many studies that have been published in reputable Chinese journals (10, 11).

Bibliometrics used in the current study is to analysis quantitatively of citation scientific publications, based on constructing the citation graph, a network representing the citations of different documents. In addition, bibliometrics is also used for exploring comprehensively the impact of their field, a set of researchers, a particular paper within a specific field of research. Furthermore, VOSviewer software was used for constructing and visualizing bibliometric networks, whereas, CiteSpace V software was utilized for visualizing co-citation networks.

There are a few published papers, using bibliometric analysis of COVID-19, to explore the activity (12) and trends (13, 14) of COVID-19 research. We aimed to explore the differences between English language and Chinese language Medical/Scientific journals publications, particularly aiming to explore the efficacy/contents of the literature published in English and Chinese in relation to the outcomes of management and characterization of COVID-19 during the early stage of COVID-19 pandemic. We have undertaken a bibliometric comparison of research on COVID-19 between English and Chinese language journals.

## Materials and Methods

### Data Source and Search Strategy

A comprehensive search was performed online using the English language databases Embase (15) and Scopus (16) on March 1, 2020, and simultaneously the Chinese databases Chinese Biomedical Database (SinoMed), CNKI, VIP and Wanfang were searched. The search terms were COVID-19, COVID 19, 2019-nCov, SARS-CoV-2, 2019 novel coronavirus, coronavirus disease 2019 and coronavirus disease-19. A detailed search strategy is presented in Supplementary Figure 1. The time period of publication was from 2019 to 1st March 2020. The search was performed on a single day to avoid bias caused by daily database updates. In the present study, only original articles and reviews published in either Chinese or English were included. The search retrieved 721 or 143 items in Chinese or English, respectively, that met the inclusion criteria.

### Eligible Criteria

In the present study, only original articles and reviews, published in the Chinese or English languages were included. Studies including the following were excluded: (1) articles or reviews published on preprint sites such as bioRiov and medRiox; (2) translated versions of articles or reviews; (3) comments, editorials, and letters; (4) eliminating duplicate literature.

### Study Selection and Data Management

Two reviewers independently performed study selection and data extraction. Differences of opinion were settled by consensus or referral to a third review author. Since some authors have the same short name, we added the affiliation behind the author names, if the same name's affiliation was different, it was considered as two different authors. For authors with more than one affiliation, we used the first one. For keywords with different expressions, we have processed them, leaving only one standardized keyword. We also reclassified publications from Hong Kong, Macau and Taiwan to China, and publications from England, Scotland, Northern Ireland, and Wales to the UK.

### Data Analysis

Publication characteristics were tabulated, including titles, authors, co-cited authors, journal sources, keywords, affiliations of authors and, for English journals, the continents, countries or regions to which the authors belong; whereas for Chinese language journals, the provinces. Co-cited authors means that the authors have been cited together. VOSviewer (version 1.6.14) software was utilized to analyze the relationships among the most highly productive countries, research institutions, and frequently used keywords. We performed cluster analysis and generated social network maps (consist of nodes and links) for countries, institutions and keywords by VOSviewer (16, 17). Cluster was also obtained by VOSviewer via analyzing the frequency of the same keywords appearing within the different papers. We set either twice or four times as the minimum frequency of keywords occurrence in English or Chinese publications, respectively, reflecting the number of included studies (143 or 721, respectively) and the consequent analysis results. Thus, the main reason for the different settings between English and Chinese is because there are more than double the number of keywords from the Chinese vs. the English language papers. Consequently, there would be too many clusters if the frequency of keywords were set as twice for the Chinese publications. Different nodes in a map represent elements including a country, institution, or keywords. The size of the nodes reflects the number of publications or frequency, the larger the node, the greater the number of publications or frequency (18). The links between nodes represent relationships of collaboration, co-occurrence, or co-citations. The color of nodes and lines represents different clusters (19). The parameters of VOSviewer were as follows: counting method (fractional counting) and “ignore documents with a large number of authors” (maximum number of authors per document is 25).

CiteSpace is scientific software that reveals the trends and dynamics in scientific literature as well as identifies key points in a given research field (18, 20). CiteSpace was therefore used to design the social network. In the current study, CiteSpace was used to identify co-occurrence maps of authors, keywords, institutions, countries or provinces and capture keywords.

## Results

A total of 864 original research articles and reviews were included, of which, 143 were retrieved from Embase and Scope in English and 721 from SinoMed, CNKI, VIP and Wanfang in Chinese.

### Authors and Journals

A total of 1,062 authors have been identified in the 143 articles published in 62 English journals. The top 10 authors and journals are listed (Table 1). The top 10 authors have contributed 46 (32.1%) of the papers. Author Li Y has the highest number of published papers (7, 4.9%), followed by Benvenuto D, Eurosurveillance Editorial Team and Leung GM (5, 3.5%), and Angeletti S, Gao GF, Ran J, Wei Y, Wu JT, and Yang G (4, 2.8%). The top 10 English journals are responsible for the publication of 72 (50.3%) papers, of which, J Med Virol is the highest (18, 12.6%), followed by Euro Surveillance (16, 11.2%) and Lancet (13, 9.1%) (Table 1).

**Table 1:** The top 10 authors and journals of COVID-19 research in English and Chinese [*n* (%)].

**Rank**	**Authors in English**	***N* (1,062, %)**	**Authors in Chinese**	***N* (3,243, %)**	**Journals in English**	***N* (143, %)**	**Journals in Chinese**	***N* (721, %)**
1	Li Y (Wuhan Uni)	7 (4.9)	Wang Y (Beijing Hosp TCM)	6 (0.8)	J Med Virol	18 (12.6)	Chin General Practice Nursing	41 (5.7)
2	Benvenuto D (Uni Campus Bio-medico of Rome)	5 (3.5)	Yang F (Tianjin Uni TCM)	6 (0.8)	Euro Surveillance	16 (11.2)	J Trad Chin Med	23 (3.2)
3	Eurosurveillance Editorial Team (European CDC)	5 (3.5)	Wang Y (Longhua Hosp, Shanghai Uni TCM)	5 (0.7)	Lancet	13 (9.1)	Chin Herb Med	18 (2.5)
4	Leung G (Uni Hong Kong)	5 (3.5)	Guo Y (1^st^ Affiliated Hosp, Xi'an Jiaotong Uni)	4 (0.6)	Emerging Microbes Infect	6 (4.2)	World J Trad Chin Med	16 (2.2)
5	Angeletti S (Univ Campus Bio-medico of Rome)	4 (2.8)	Lei X (^2nd^ Affiliated Hosp, Xi'an Jiaotong Uni)	4 (0.6)	NEJM.	6 (4.2)	Herald Med	15 (2.1)
6	Gao G (China CDC)	4 (2.8)	Li S (^2nd^ Affiliated Hosp, Xi'an Jiaotong Uni)	4 (0.6)	Viruses	5 (3.5)	Shanghai Med J	14 (1.9)
7	Ran J (Uni Hong Kong)	4 (2.8)	Luo F (Union West China Hosp, Sichuan Uni)	4 (0.6)	Radiology	4 (2.8)	Chin J Tuberc Resp Dis	13 (1.8)
8	Wei Y (Wuhan Jinyintan Hosp)	4 (2.8)	Miao Q (Xiyuan Hosp, Academy Chinese Med Sci)	4 (0.6)	World J Pediatric	4 (2.8)	Chin J Prev Med	12 (1.7)
9	Wu J (Uni HongKong)	4 (2.8)	Shi J (Longhua Hosp, Shanghai Uni TCM	4 (0.6)	BML	3 (2.1)	Chongqing Med	11 (1.5)
10	Yang G (Chinese Uni Hong Kong)	4 (2.8)	Shu B (Longhua Hosp, Shanghai Uni TCM)	4 (0.6)	JAMA	3 (2.1)	China Trop Med/ Chin J Resp Critical Care Med/Chin Nur Res	10 (1.4)

Meanwhile, 3,243 authors have been identified in the 721 articles published in 193 Chinese journals. The top 10 authors have contributed 45 (6.2%) of the papers. Authors Wang YG and Yang FW have published the highest number of papers (6, 0.8%), followed by Wang YJ (5, 6.9%). The top 10 Chinese journals have published 193 (26.8%) of the papers, the highest is Chinese General Practice Nursing (41, 5.7%), followed by J Traditional Chin Med (23, 3.2%) and Chin Herb Med (18, 2.5%) (Table 1).

**Figure 1:**
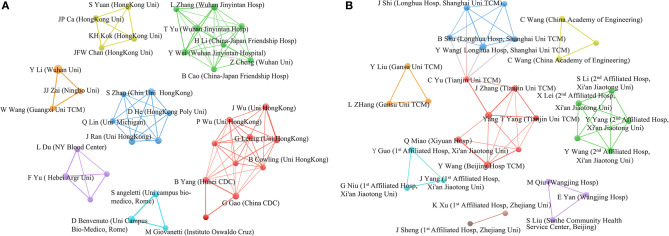
The level of cooperation among the identified authors. **(A)** for English papers; **(B)** for Chinese papers.

For the analysis of the social relationships of authors (affiliated institutions) with more than three articles (Figure 1), it was found that of 38 authors who published English papers, seven clusters corresponding to seven categories were identified (A), and of 29 authors who published Chinese papers, clustering identified eight categories (B). These categories demonstrate that the cooperation between the various authors is close.

### Countries/Provinces (Areas) and Institutions

**Figure 2:**
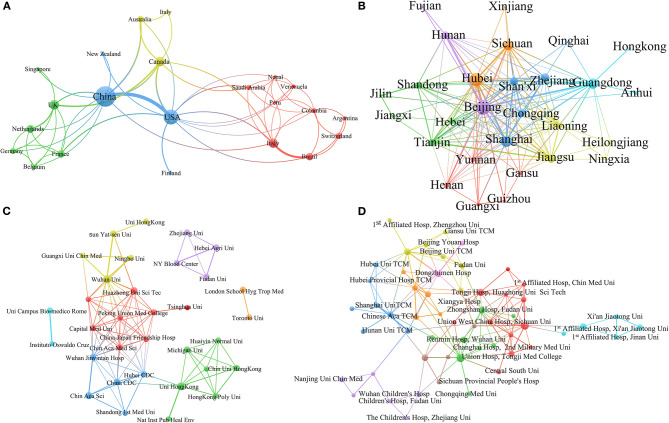
**(A)** The cooperation among the countries **(A)** and provinces in China **(B)**; The cooperation among the institutions for English **(C)** and Chinese **(D)** publications.

Of a total of 143 English papers that were published, there were a total of 1,062 authors from 32 countries or areas, including China (75/143, 52%), USA (34/143, 24%), UK (11/143, 8%), Canada (11/143, 8%), and Italy (10/143, 7%). There are 252 institutions from 32 countries published COVID-19 related English papers. The first five are from China, including Wuhan University (15/252, 6%), University of Hong Kong (12/252, 5%), Chinese Academy of Sciences (9/252, 4%), Huazhong University of Science and Technology (8/252, 3%), and Chinese CDC (6/252, 2%). For the analysis of the social relationships of countries with more than three articles (Figure 2A), 22/32 countries are clustered into four categories; 33/35 institutions are clustered into seven categories (Figure 2B), demonstrating the cooperation between countries and institutions is close.

Meanwhile, within the Chinese literature, of the 721 papers published there are 3,243 authors from 30 Provinces, autonomous regions or municipalities in China who contributed to the publications. The top five are Beijing (168, 23%), Hubei (136, 19%), Shanghai (90, 12%), Guangdong (78, 11%), and Sichuan (70, 10%). However, the provinces/Autonomous Regions who published the lowest number of articles in Chinese include Xinjiang Uygur, Qinghai and Ningxia, Inner Mongolia and Hong Kong, had 2 articles each. There are 677 institutions who published COVID-19 related papers. The institutions that have published the largest number of papers in Chinese are the Union Hospital, Huazhong University of Science & Technology (37, 5%), West China Union Hospital, Sichuan University (32, 5%), Tongji Hospital, Huazhong University of Science & Technology (23, 3%), Beijing University of Chinese Medicine (18, 3%), Zhongnan Hospital, Wuhan University (16, 2%) (Table 2).

**Table 2:** The top five countries/areas and institutions that contributed to publications of COVID-19 research in English and Chinese [*n* (%)].

**Rank**	**Countries/areas**	***N* (%)**	**Institution**	***N* (%)**
**COUNTRIES IN ENGLISH JOURNALS**				
1	China	75 (52)	Wuhan University, Hubei	15 (6)
2	USA	34 (24)	University of HongKong	12 (5)
3	UK	11 (8)	Chinese Academy of Sciences	9 (4)
4	Canada	11 (8)	Huazhong Uni Sci Technol, Hubei	8 (3)
5	Italy	10 (7)	Chinese CDC	6 (2)
**PROVINCES IN CHINESE JOURNALS**				
1	Beijing	168 (23)	Union Hosp, Tongji Med College, Huazhong Uni Sci Technol, Hubei	37 (5)
2	Hubei	136 (19)	Union West China Hosp, Sichuan Uni	32 (5)
3	Shanghai	90 (12)	Tongji Hosp, Tongji Med College, Huazhong Uni Sci Technol, Hubei	23 (3)
4	Guangdong	78 (11)	Beijing Uni Chin Med	18 (3)
5	Sichuan	70 (10)	Zhongnan Hosp, Wuhan Uni, Hubei	16 (2)

For the analysis of the social relationships of provinces/areas with more than three articles, as can be seen from Figure 2C, amongst 32 provinces/areas, 28 provinces/areas are clustered into seven categories; amongst 677 institutions, 56 are clustered into nine categories, and the cooperation between them is close with more than three articles (Figure 2D).

### Co-occurrence of Keywords

**Figure 3:**
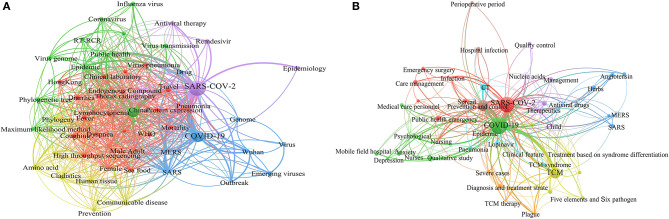
The cluster analysis of English **(A)** and Chinese **(B)** keywords.

For the papers published in English, 471 English keywords are extracted from the 143 articles. A density map is generated for keywords with a co-occurrence greater than twice, including 54 keywords in the map (Figure 3A). SARS-CoV-2 was the most frequently used keyword (Figure 3A), with 93 (19.7%) co-occurrences, followed by COVID-19 (44, 9.3%), China (36, 7.6%), SARS (22, 4.8%), and epidemic (17, 3.6%) (Table 3). Among the top 20 keywords, some are related to epidemiological characteristics, such as epidemic, adult, male, female, travel, others are related to a comparison with similar diseases, e.g., MERS, SARS. Some are correlated to the structure of the virus, e.g., endogenous compound, amino acid, cladistics, and phylogeny. Cluster analysis is performed on co-occurrence of English keywords with a frequency >2. There are 54 keywords clustered into five categories (Supplementary Figure 1B). Cluster 1 includes 22 keywords, adult, clinical feature, clinical laboratory, coughing, diarrhea, female, fever, gene expression, high throughput sequence, Hong Kong, intensive care unit, lymphocytopenia, male, mortality, pneumonia, protein expression, sea food, thorax radiography, travel, virus pneumonia and World Health Organization; Cluster 2 mainly focuses on China, coronavirus, epidemic, influenza virus, phylogenetic tree, public health, reverse transcription polymerase chain reaction, virus detection, virus genome, virus transmission; Cluster 3 mainly focuses on COVID-19, drug, emerging virus, genome, MERS, outbreak, SARS, virus and Wuhan.

**Table 3:** The top 20 keywords in terms of frequency for COVID-19 research in English and Chinese [*n* (%)].

**Rank**	**English keywords**	**N (471, %)**	**Chinese keywords**	***N* (1,234, %)**
1	SARS-CoV-2	93 (19.7)	COVID-19	543 (44.0)
2	COVID-19	44 (9.3)	SARS-CoV-2	381 (30.9)
3	China	36 (7.6)	Prevention and control	141 (11.4)
4	SARS	22 (4.8)	Traditional Chinese Medicine	140 (11.3)
5	Epidemic	17 (3.6)	Computed tomography	35 (2.8)
6	Adult	15 (3.2)	Epidemic	29 (2.4)
7	Psychological	15 (3.2)	Public health	24 (1.9)
8	Nucleic acids	12 (2.5)	MERS	21 (1.7)
9	Plague	11 (2.3)	Pneumonia	21 (1.7)
10	Infection	11 (2.3)	Male	20 (1.6)
11	Child	8 (1.7)	Wuhan	19 (1.6)
12	Antiviral drugs	8 (1.7)	Endogenous compound	18 (1.5)
13	Nursing	8 (1.7)	Female	18 (1.5)
14	Therapeutics	6 (1.3)	Phylogeny	15 (1.5)
15	Diagnosis	5 (1.1)	Outbreak	14 (1.2)
16	MERS	5 (1.1)	Communicable disease	13 (1.1)
17	Clinical feature	5 (1.1)	Fever	13 (1.1)
18	Nurses	4 (0.9)	Travel	13 (1.1)
19	Amino acid	4 (0.9)	Traditional Chinese Medicine therapy	13 (1.1)
20	Angiotensin	4 (0.9)	Cladistics	9 (0.9)

A total of 1,234 Chinese keywords are extracted from the 721 Chinese-language articles. A density map is generated for keywords with a co-occurrence >4 times, resulting in the generation of five categories (Table 3). As stated above, there are substantial more Chinese keywords identified within the Chinese Journals. If thence-occurrence of three times or less is adopted for the analysis, the clusters would be too many to offer an objective outcome. COVID-19 is the most frequently used keyword, with 543 (44.0%) co-occurrence (Figure 3B), followed by SARS-CoV-2 (381, 30.9%), TCM (153, 12.4%), prevention and control (141, 11.4%), epidemic (56, 4.5%), management (51, 4.1%), therapeutics (48, 3.9%), and computed tomography (CT) (35, 2.8%). Among the selected top 30 keywords with frequency more than 10, there were five clusters generated with such information (Figure 3B). For more detailed clusters, these were as follows: The keywords from the cluster one included clinical symptoms, critical case, CT, diagnosis, nucleic acids, therapeutics, X-ray; the keywords from the cluster two included cancer patients, emergency, infection, management, medical care personnel, mental health, prevention and control; the keywords for the cluster three were Covid-19, epidemic, guidelines, integrated Chinese & Western medicine, TCM, the scheme of diagnosis and treatment, treatment based on syndrome differentiation; the cluster four included angiotensin, antiviral drugs, herbs, MERS, SARS-COV-2; finally the cluster five were nursing and pregnant.

### Network Social Analysis Between China and Other Countries

There are total 143 papers written in English within this study from which data has been collected. Of these 143 papers, 21 articles were written jointly between Chinese authors and authors from other countries, while 54 papers published in English were authored by Chinese authors only. Among the 21 articles authored jointly, the order of co-operations was: China and USA (14/21), China and UK (3/21), and then Australia and Canada (2/21). To demonstrate this point, a network social analysis was performed (Figure 2A).

For the 21 English language articles jointly authored between Chinese and international authors, the institutions involved in cooperation between China and other countries were found to be centered in Hong Kong, Hubei Province, Beijing and Shanghai within China, while the most frequent overseas institution involved in cooperation was the New York Blood Center from the USA (Figure 2C). This cooperation covered a range of scientific topics, mainly focusing on diagnosis, such as PCR testing in the laboratory, prevention and control, and the viral genome (Figure 3A).

## Discussion

The battle against COVID-19 has been highly effective in China up to date, however, the pandemic of COVID-19 is highly alarming in around the world with substantial morbidity and mortality (3). The most urgent task for medical doctors/scientists is to control COVID-19, including the incorporation of aspects of the Chinese approaches. Many diverse studies addressing COVID-19 have sprung up due to the urgent necessity of prevention and control.

We have focused on English and Chinese publications only for the comparison. Most of the studies captured in this paper on COVID-19 in English journals have been conducted by Chinese scholars and institutions, which is highly likely to be due to the timing of the literature search for this study, March 1st 2020, at which point the predominantly affected locations were Wuhan, and to a lesser extent the remainder of China. Of the international publications, particularly from Western countries, e.g., Italy (21) and South Korea (22), these publications occurred during the latter part of the survey period, from 27 February 2020, which is likely to be attributed to the spread of the SARS-CoV-2 commencing within these other countries, both raising the index of concern within those other regions and directly making available to those regions affected local populations and biological materials on which studies could be conducted. More authors from Hong Kong published more English papers than papers in Chinese, which may be due to the higher levels of advanced English literacy, reflecting the English-based educational system (23). Furthermore, the majority of the Hong Kong researchers have more opportunity to study/work and establish links overseas (24), in addition to their preference for English journals. Although the impact of COVID-19 in Iran has been very severe (25), there has been no studies published on the pandemic at all prior to March 1st, 2020. We speculate that the Iranian government has experienced difficulties scaling up its response to combating the epidemic, due to the economic loss and supply issues associated with economic sanctions imposed (26).

Similarly, the scholars who published studies on COVID-19 in Chinese journals are mainly from Beijing, Hubei, Shanghai, Guangdong and Sichuan. A likely explanation is that most of the first-class medical universities are within these areas, corresponding to the top research institutional distribution in China. Apart from Wuhan, Hubei Province, Sichuan University has published more papers than other areas, except for Beijing, Shanghai and Guangdong, which are the three provinces with the highest GDP in China (27). Especially relevantly, as the capital of China, Beijing is the nation's political, economic, cultural and educational center, and has the largest number of universities in the country. These data support the idea that advanced academic development needs financial support. Certainly, less publications are from Xinjiang Uygur, Qinghai, Ningxia, and Inner Mongolia, all of which has fewer COVID-19 cases, but also have lower GDP within remote northwest China (GDP rankings out of 31 regions 19, 23, 15, 9, respectively) (28). In the cluster of authors, we found that the cooperation between the various authors is close but there is not a hotspot amongst them, which is in line with the reality that the information sharing was lacking at the early stage.

The studies in English related to COVID-19 are published in international, highly reputable journals, including Nature, Science, Cell, NEJM, JAMA, and Lancet. These publications enable medical practitioners/scientists to share/exchange information efficiently, providing essential background for some key policy decisions (29, 30), e.g., mandatory wearing of face masks, minimizing social gathering [has been widely accepted, including Australia (31), UK (32)], and the lockdown of interstate travel in many countries of the EU (33). Importantly, the ultra-rapid development of an effective vaccine, has been accelerated by the rapid sharing of scientific data, particularly the published sequences of the SARS-CoV-2 virus. Thus, publications in English journals, particularly in well-recognized, top ranking international journals, results in rapid dissemination of key information for use of the data for practical applications. English is the well-accepted communication language of science around the world.

Our data demonstrated that substantial collaborative research has been undertaken from the very early period of the COVID-19 outbreak (34, 35), and that this research has become more frequent and deep following the declaration of a pandemic. Such collaborations are certainly enhancing our understanding of the nature of the SARS-CoV-2 virus (36, 37), have supported development of effective vaccines (38, 39), and has provided vital data to assist clinical diagnosis at the international level (40). These developments further support our conclusion that publications in English have enabled doctors/scientists to effectively share/exchange information at the international level. The cluster analysis of institutions at the international level demonstrated strong regional representation even at the international level, both within China and within international countries. Interestingly, the cluster of cooperation for studies in China was thickest with the USA, suggesting the cooperation was mainly between China and USA, which is consistent with the publications retrieved from the database. The most likely explanation has been mentioned above, namely that the economic resources of each country is the likely most significant factor to impact both the disease and research into it.

In contrast, there is a language barrier to the utilization of the information from the papers published in English journals for use by the general population in China. The publications in the Chinese language are able to meet a complementary dissemination purpose for China-based medical practitioners to understand the essential key information concerning COVID-19, especially for those in the remote areas of China, without proper access of English journals or sufficient language skills (41). Indeed, publications in Chinese provide a more acceptable approach for Chinese doctors to learn how to deal with COVID-19 in the relatively remote regions of China, an outcome that is consistent with the large number of studies that have been published in Chinese.

The top 10 Chinese journals that included COVID-19-related papers are mostly from the Chinese Science Citation Database, representing the most authoritative and representative core of journals in all disciplines in China (42). Importantly, in this study there are a total of 721 papers that cover COVID-19 from various scientific areas within the identified journals, often with a large number of authors, reflecting the Chinese authority's intention to accelerate the control of COVID-19 and the rapid dissemination of knowledge.

There are a total 471 or 1,234 keywords in English or in Chinese publications, respectively, used in the studies on COVID-19 that we identified till March 1st, 2020. However, more than 78% of the keywords appeared once, only 3.9% of the English keywords have a frequency of >4, indicating the importance of a few keywords. In bibliometrics, a network graph of keyword co-occurrences reflects hot topics (18). Cluster analysis of co-occurrence keywords demonstrates that there are five clusters in this field. Cluster 1 consists of 22 keywords, mainly relates to the epidemiological characteristics and clinical features, because these are the basis for understanding key aspects of the disease, such as treatment and control. At the present time, many scholars are focusing on the large proportion of COVID-19 patients who exhibit mild symptoms or are asymptomatic carriers, reflecting the seriousness of the nature of viral transmission (43). Cluster 2 contains 11 keywords, mainly focuses on the virus detection and genome. Some data demonstrate that bat CoV and human SARS-CoV-2 might share the same ancestor (40), and similar residues of the key receptor are observed in many species (44). Because of the importance of the original source of SARS-CoV-2, the evolution and genomics is a hot topic in this field. Nine keywords are included in cluster 3, focusing on drug treatment and comparison with SARS and MERS, making comparisons to these fatal respiratory tract infections by coronaviridae, to explore any clues between the similarity and differentiation.

For the papers published in Chinese language journals, there are five clusters of keywords, including 2–6 keywords in each field. Cluster 1 consists of six keywords, mainly relating to treatment and diagnosis, because these activities are the basis for understanding key aspects of the disease, such as treatment and control. At the present time, many scholars are focusing on the large proportion of COVID-19 patients who exhibit mild symptoms or are asymptomatic carriers, reflecting the seriousness of the nature of viral transmission (43). Cluster 2, contains 6 keywords, mainly focusing on emergency, infection, management, medical carers, prevention and control, which are supported by the others, demonstrating the critically importance of COVID-19 in such outbreak (45), transmission (46) and disease control and management (47). Cluster 3 is focusing on TCM or Chinese and Western treatment for COVID-19, mainly to explore the benefit of the combination of TCM and classical Western management approaches, especially aiming to provide the guidelines for relatively remote/rural regions of China. The advantage of this particular cluster is its usefulness in the outskirts of metropolitan or rural areas, where there is a relative lack of advanced or first line anti-viral medications (48). Cluster 4 is an extension of the current existing treatment to the cardiovascular system (49), as well as, using the previous experience in MERS (50), and also places emphasis on anti-viral drugs and herbs (51). Interestingly, cluster 5 includes pregnancy and nursing, which is a very venerable population at high risk, either due to compromised immunity during pregnancy (52) or the lack of sufficient data to adequately understand the severity of the potential risk of COVID-19 in pregnancy and the need to guard against COVID-19 infection in pregnancy (53).

Part of the reason for the Chinese scholars focus on TCM when publishing in Chinese medical journals is the difficulty Chinese scholars have to disseminate their findings using modern scientific terminology/theory, compared to rather ancient theory of TCM, e.g., balance of Ying and Yang. Actually, we believe that balance of Ying and Yang is equivalent to the modern theory of anti-vs. pro-inflammatory responses in the micro-environment, i.e., imbalance of anti-vs. pro-inflammatory responses contributes to autoimmune diseases (54). Thus, from the point of view of the management of COVID-19, the efforts should be focused on the suppression of the SARS-CoV-2 virus, disregarding the backgrounds, theories, and approaches of modern vs. traditional scientific ideology. Consequently, analysis of the dissemination of the critical information from English and Chinese languages could facilitate such a purpose appropriately.

These COVID-19 related English language papers, especially at the top end, e.g., NEJM (55), Lancet (7, 26), Science (8), Nature (36), provide the most critical information in the development of effective vaccinations (31). On the other hand, for many primary health carers at the front line in the relatively remote regions in China, obtaining the most up dated information of COVID-19 particularly, regarding prevention and/or controlling has been from the Chinese language. In addition, the local government at the county levels are also heavily dependent on such key information in Chinese, in detail, e.g., keep social distance, no public gathering, and lockdown of manufacture and so on (56).

There are some limitations in the current study. First, our study is focusing only on English and Chinese journals, which inevitably could miss some important information from other languages. We will further analyze such points by collaborating with researchers from the different regions/countries. Second, our study has been undertaken at the vortex of the epidemic before March, 2020, which may miss the most updated information. Third, the total number of included studies is relatively small, and the study duration of just more than 2 months from when the first COVID-19 patient is identified till March 1st, 2020, is a short cutoff time for data retrieval.

## Conclusions

The publications related to COVID-19 research has been rapidly growing since the disease emerged. More studies have been published in Chinese journals than in English, due to the epicenter being located in Wuhan, China before March 1st 2020. The publications in English have enabled doctors/scientists to share/exchange information at the international level; the publications in the Chinese language provides complementary educational approaches for the local doctors to understand the essential and key information to manage COVID-19 in the relatively remote regions of China for the general population.

## Data Availability Statement

The raw data supporting the conclusions of this article will be made available by the authors, without undue reservation.

## Author Contributions

JF and SB conceived of the presented idea. JF and YG developed the theory and performed the computations. YG and JT verified the analytical methods. NZ, RD, HZ, XF, and GS collected and synthesized the data. CC and BH encouraged JF and YG to investigate and supervised the findings of this work. All authors discussed the results and contributed to the final manuscript.

## Conflict of Interest

The authors declare that the research was conducted in the absence of any commercial or financial relationships that could be construed as a potential conflict of interest.

## Supplementary Material

Supplementary Figure 1 The density map of English **(A)** and Chinese **(B)** keywords for COVID-19 publications.
