# Supplementary figures and images for: Bibliometric Analysis on COVID-19: A Comparison of Research Between English and Chinese Studies
Source: Front Public Health. 2020 Aug 14;8:477. doi: 10.3389/fpubh.2020.00477 (PMC7456831; doi:10.3389/fpubh.2020.00477)

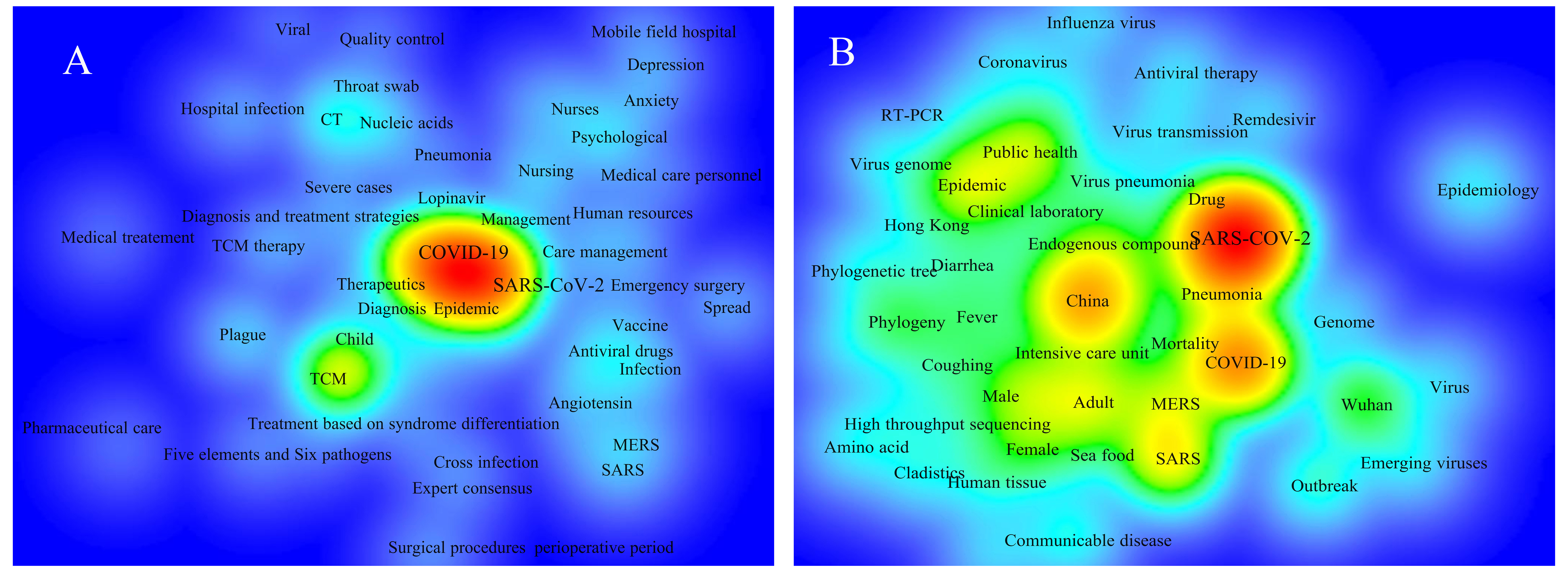

Supplement: Supplementary Figure 1 — The density map of English (A) and Chinese (B) keywords for COVID-19 publications. [file Image_1.jpg]
